# The antennal transcriptome analysis and characterizations of odorant-binding proteins in *Megachile saussurei* (Hymenoptera, Megachilidae)

**DOI:** 10.1186/s12864-023-09871-8

**Published:** 2023-12-15

**Authors:** Wei-Zhen Li, Wen-Juan Kang, Jing-Jiang Zhou, Su-Qin Shang, Shang-Li Shi

**Affiliations:** 1 Key Laboratory of Grassland Ecosystem of Ministry of Education, and Sino-U.S. Centers for Grazingland Ecosystem Sustainability, College of Grassland Science, Gansu Agricultural University, Lanzhou, 730070 China; 2 College of Plant Protection, Biocontrol Engineering Laboratory of Crop Diseases and Pests of Gansu Province, Gansu Agricultural University, Lanzhou, 730070 China; 3 Department of Biological Chemistry, Rothamsted Research, Harpenden, Hertfordshire UK

**Keywords:** *Megachile saussurei*, *Medicago sativa*, Odorant-binding proteins, Transcriptome, Antennae

## Abstract

**Background:**

Odorant-binding proteins (OBPs) are essential in insect’s daily behaviors mediated by olfactory perception. *Megachile saussurei* Radoszkowski (Hymenoptera, Megachilidae) is a principal insect pollinating alfalfa (*Medicago sativa*) in Northwestern China. The olfactory function have been less conducted, which provides a lot of possibilities for our research.

**Results:**

Our results showed that 20 OBPs were identified in total. Multiple sequence alignment analysis indicated MsauOBPs were highly conserved with a 6-cysteine motif pattern and all belonged to the classic subfamily, coding 113-196 amino acids and sharing 41.32%-99.12% amino acid identity with known OBPs of other bees. Phylogenetic analysis indicated there were certain homologies existed among MsauOBPs and most sequences were clustered with that of *Osmia cornuta* (Hymenoptera, Megachilidae). Expression analysis showed the identified OBPs were mostly enriched in antennae instead of other four body parts, especially the MsauOBP2, MsauOBP3, MsauOBP4, MsauOBP8, MsauOBP11 and MsauOBP17, in which the MsauOBP2, MsauOBP4 and MsauOBP8 presented obvious tissue-biased expression pattern. Molecular docking results indicated MsauOBP4 might be the most significant protein in recognizing alfalfa flower volatile 3-Octanone, while MsauOBP13 might be the most crucial protein identifying (Z)-3-hexenyl acetate. It was also found the lysine was a momentous hydrophilic amino acid in docking simulations.

**Conclusion:**

In this study, we identified and analyzed 20 OBPs of *M. saussurei*. The certain homology existed among these OBPs, while some degree of divergence could also be noticed, indicating the complex functions that different MsauOBPs performed. Besides, the *M. saussurei* and *Osmia cornuta* were very likely to share similar physiological functions as most of their OBPs were clustered together. MsauOBP4 might be the key protein in recognizing 3-Octanone, while MsauOBP13 might be the key protein in binding (Z)-3-hexenyl acetate. These two proteins might contribute to the alfalfa-locating during the pollination process. The relevant results may help determine the highly specific and effective attractants for *M. saussurei* in alfalfa pollination and reveal the molecular mechanism of odor-evoked pollinating behavior between these two species.

**Supplementary Information:**

The online version contains supplementary material available at 10.1186/s12864-023-09871-8.

## Background

For a long period, insects have gradually adapted to the complex and ever-changing physiological environment with their sensitive olfactory system recognizing a large number of odor chemicals, which plays a crucial role in host selection, feeding, mating, and reproduction [1–3]. Insect’s antenna, covered by multi-type olfactory sensilla like the basiconic, coeloconic, and trichoid, is the central organ in sensing and recognizing external odors [4]. The sensilla are filled with potassium- and protein-rich fluid called sensillum lymph, which bathes the dendrites [5, 6]. Many chemosensation-related proteins secreted in sensillum lymph are involved in the complex olfactory-perception process, such as Odorant-binding proteins (OBPs), odorant receptors (ORs), chemosensory proteins (CSPs), ionotropic receptors (IRs), sensory neuron membrane proteins (SNMPs), and odorant-degrading enzymes (ODEs) [7, 8].

Among all those olfaction-related proteins, OBPs function as the initial step in odorant recognition and transduction [9, 10]. OBPs were a group of small, soluble, and acidic proteins with a highly-conserved structure [11, 12]. Generally, OBPs are classified into five diverse subtypes based on the number and model of conserved cysteines in their amino acid sequence [13], which includes Classical OBPs (those with 6 conserved cysteines), Minus-C OBPs (those with only 4 conserved cysteines), Plus-C OBPs (those with 8 conserved cysteines), dimer OBPs (those with 12 conserved cysteines) and Atypical OBPs (those with 9~10 conserved cysteines) [14, 15]. Upon encountering external chemical signals, such as pheromones, plant volatiles or odors from other species, odor molecules would enter the sensillum lymph through the massive pores on the sensilla, and OBPs in the lymph immediately recognize, bind and shift the newly-formed odor-OBP complexes to the ORs in sensory dendrites, which transform the chemical signals to electrophysiological signals and eventually trigger the corresponding behavior of insects [16–18].

OBPs have been intensively studied since the first report in a moth, *Antheraea polyphemus* [19]. Various OBPs and multiple functions accordingly have been identified. A class of GOBPs binding and transporting common odor molecules in the antennae of female *Antheraea pernyi* were identified [20] (Breer et al., 1990). Biochemical binding kinetics studies found the dual role of transporting and inactivating odorous substances [21, 22]. A study of *Drosophila melanogaster* mutants showed that OBPs are involved in the transport of odor molecules to ORs [23]. Besides, ApisOBP3 in *Acyrthosiphon pisum* [24], GmolGOBP2 in *Grapholita molesta* [25] and OBP6 in *Meteorus pulchricornis* [26] all demonstrated that OBPs could specifically recognize and screen specific chemical signals. Recently, The rapid development of techniques like electrophysiology, RNA interference, and gene knockout has directly revealed the necessity of OBPs for proper functioning in the olfactory system [27–30].

Information on three-dimensional structures and binding modes can elucidate the critical functions of the soluble olfactory proteins in insects’ daily behavior [31]. The interaction between OBPs and ligands based on molecular docking method has been widely conducted and the 3D-structures of over 20 OBPs in different insect species were reported including Diptera, Hemiptera, and Lepidoptera, etc. [2, 8]. Not only the OBPs, other soluble olfactory proteins such as CSPs were also studied using the molecular docking method. Previous studies even pointed out that molecular docking could function as a tool for screening the best ligands for insects [32]. These examples demonstrated that this virtual method has been reliable in the olfactory study of insects. To date, no such research has been conducted against MsauOBPs. Knowledge of the interaction of specific alfalfa flower volatile and MsauOBPs is still deficient.

*M. saussurei* is a principal pollinator of alfalfa (*Medicago sativa* L.) in Yumen area, Gansu province, which is one of the most important bases cultivating alfalfa in Northwest of China. Unlike the most intensively managed and studied alfalfa leaf-cutting bee (*Megachile rotundata*) [33] and other commercially produced bees, information in many aspects has been little known about this species. The objective of this study was to identify the odorant-binding proteins in female *M. saussurei* based on antenna-specific transcriptome analysis. Because males would die soon after they copulate with females [34], indicating female *M. saussurei* are the main force pollinating alfalfa. In this study, the antennae transcriptome sequencing of *M. saussurei* was performed and we also compared the putative OBPs in *M. saussurei* with those from other bees using phylogenetic analysis and determined the type of OBPs. The quantitative real-time PCR was thereafter applied to verify the expression pattern and level in five different tissues of *M. saussurei*. Finally, the interaction of MsauOBPs of two alfalfa flower volatiles was simulated using the molecular docking method. This is the first research investigating olfaction-related genes against *M. saussurei*, by which promising insights into the molecular mechanism of odor-evoked pollinating behavior and the development of highly specific and effective attractants for alfalfa pollination might be provided.

## Results

### Antenna transcriptome sequencing

In this research, three RNA-seq libraries were constructed, and a total of 45,573,892 raw reads were obtained in each sample. After removing reads containing over 5% unknown bases (971,132 in average), those containing adapters (1,579,565 on average) and those of low quality (40 on average), 43,023,155 clean reads on average were obtained, which accounts for 94.40% in total raw reads. The Q30 content in each sample was >90% (Table S3).

We obtained the unigenes with Trinity assembling followed by Tgicl-clustering in three samples. To improve the reliability of the assembly, Tgicl was used again to cluster the initial unigenes from three samples and finally generated the “all-unigene” assembly, which was used in the following analysis (Table S4). We acquired 77,444 unigenes in total with a total length of 184,461,623 bp and a mean length of 2381 bp. The values of N50, N70, and N90 were 4540 bp, 2,951 bp, and 1,263 bp respectively, and the GC content was 38.03% (Table S4). The number of unigene sequence sizes between 200-300 bp, 300-400 bp, and more than 3000 bp were 12041, 5779, and 22901 respectively, while the number of sequence sizes between 400-3000 bp was all lower than 5000 bp (Fig. S2). Results on assembly evaluation indicated only a small number of sequences were fragmented and missed in three samples and all-unigene, while more than 95% were able to match the sequences in the BUSCO database (Fig. S3), which indicated our unigenes were well assembled.

### Functional annotation of unigenes

The 77,444 unigenes were functionally annotated in seven publicly accessed databases, among which 53,991 in NR (69.72%), 63,871 in NT (82.47%), 42,868 in SwissProt (55.35%), 42,052 in KOG (54.30%), 47,037 in KEGG (60.74%), 17,258 in GO (22.28%), and 43,002 in Pfam (55.53%) were successfully annotated, respectively (Table S5).

**Fig. 1:**
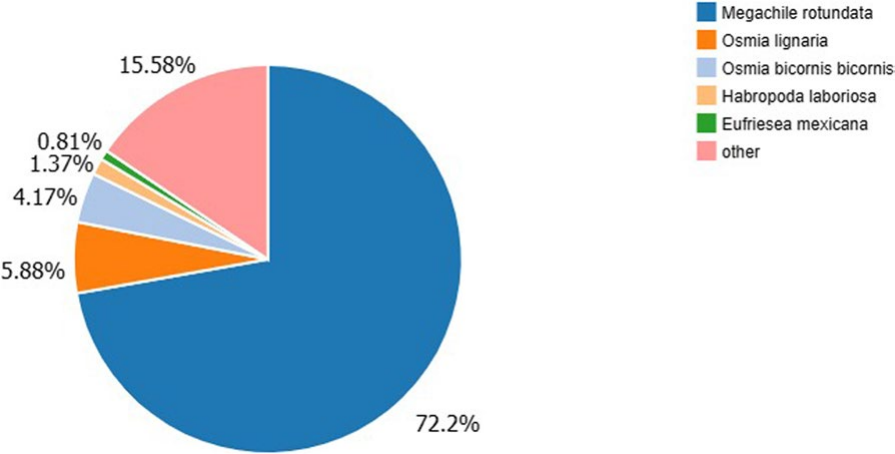
Species classification of best blast hit in transcriptome analysis

**Fig. 2:**
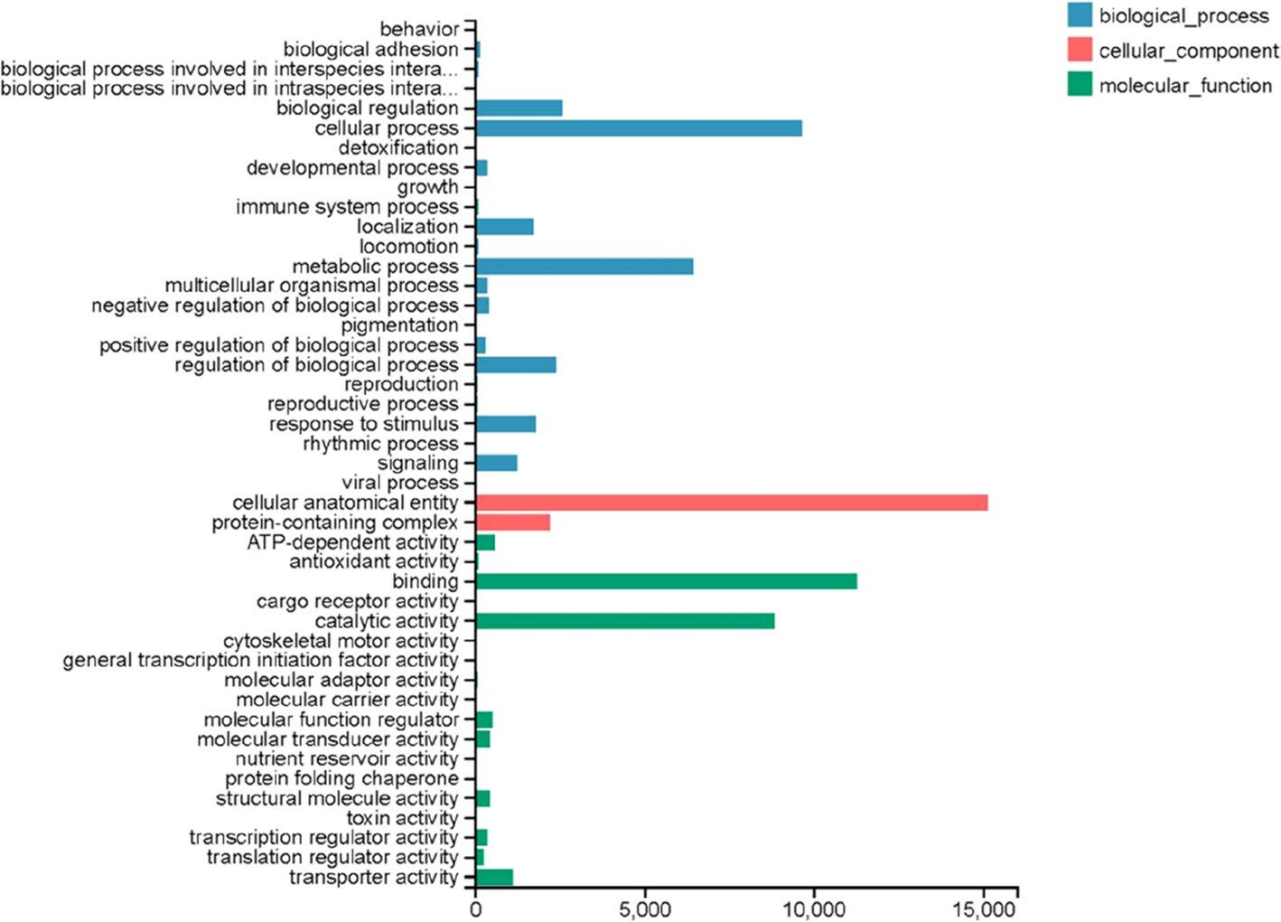
GO enrichment analysis of *M. saussurei* unigenes

**Fig. 3:**
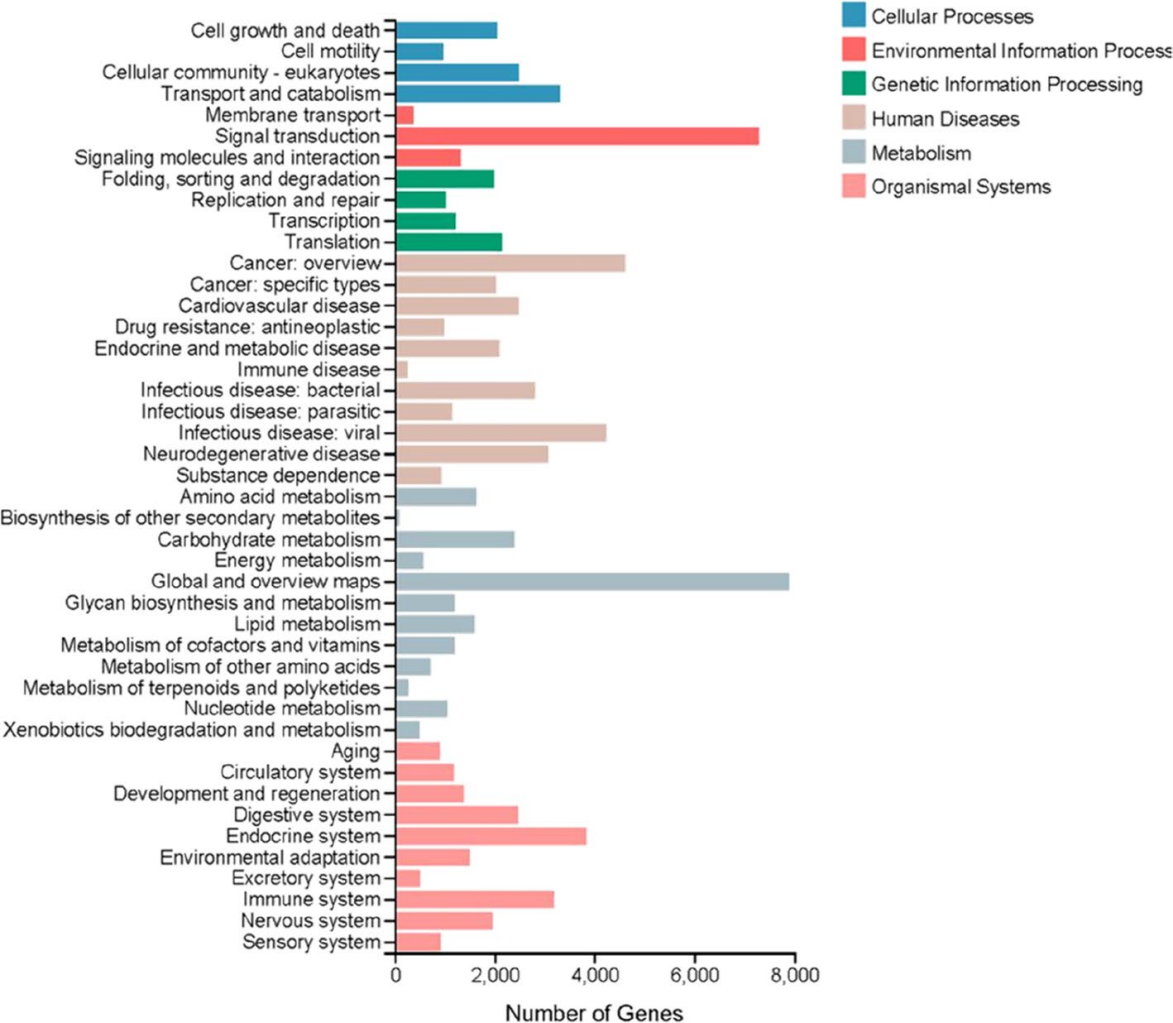
KEGG pathway analysis of *M. saussurei* unigenes

Figure 1 indicated that 72.20% of the *M. saussurei* unigenes annotated in NR database have best hits with genes in *M. rotundata*, followed by *Osmia lignaria* (5.88%) and *Osmia bicornis* (4.17%) (Fig. 1). Three functional categories, biological process, cellular component, and molecular function, were annotated in GO annotation (Fig. 2). In the biological process category, the genes expressed in the antennae were mostly enriched to the cellular process and metabolic process. In the molecular function category, binding and catalytic activity accounted for more than 80% of the total unigenes, while only two terms existed in the cellular component category, namely cellular anatomical entity and protein-containing complex. In KEGG analysis, unigenes were assigned and annotated into six different pathways, cellular processes, environmental information processing, genetic information processing, human diseases, metabolism, and organismal systems. The specific term “signal transduction” with 7286 unigenes in the environmental information processing pathway was closely related to odorant binding proteins, having the second largest number of unigenes (Fig. 3).

### Identification of odorant-binding protein genes

Based on the highly conserved amino acid sequence structure, we identified a total of 20 OBP genes from the *M. saussurei* antennae transcriptome and named them after MsauOBP1-5 and MsauOBP7-21. Detailed information on these OBPs is displayed in Table 1. All sequences contained complete open reading frame (ORF), coded 113-196 amino acids, and shared 41.32%-99.12% amino acid identity with known OBPs of other bees. Most OBPs contained a N-terminal signal peptide except MsauOBP1, 5, 7, 9, 12, 21. All sequences have been uploaded to GenBank, the accession number and other best blast match results can also be seen in Table 1.

**Table 1:** The sequence information of 20 identified odorant-binding proteins in *M. saussurei* antenna

Gene name	Accession No.	ORF (aa)	Signal peptide	Complete ORF	Best blast match				
					Species	Acc. number	ORF(aa)	E value	Identity
*MsauOBP1*	OR266110	140	1-22	Yes	*Osmia lignaria*	XP_034185749.1	141	1e-56	59.57%
*MsauOBP2*	OR266111	133	1-17	Yes	*Megachile rotundata*	XP_003708550.1	133	1e-82	89.47**%**
*MsauOBP3*	OR266112	133	1-21	Yes	*Megachile rotundata*	XP_003708550.1	133	1e-88	96.99**%**
*MsauOBP4*	OR266113	139	1-19	Yes	*Osmia cornuta*	AGI05203.1	137	4e-37	52.94%
*MsauOBP5*	OR266114	113	No	Yes	*Osmia cornuta*	AGI05203.1	137	7e-23	49.09%
*MsauOBP7*	OR266116	146	1-24	Yes	*Apis mellifera caucasica*	KAG6801071.1	131	5e-43	63.03%
*MsauOBP8*	OR266117	143	1-19	Yes	*Osmia cornuta*	AGI05200.1	143	2e-68	65.03%
*MsauOBP9*^a^	OR266118	117	1-19	Yes	*Megachile rotundata*	XP_012149308.1	166	1e-78	100%
*MsauOBP10*	OR266119	150	1-23	Yes	*Bombus terrestris*	XP_003398556.1	152	2e-81	95.31%
*MsauOBP11*	OR266120	142	1-21	Yes	*Osmia cornuta*	AGI05201.1	142	1e-59	66.90%
*MsauOBP12*	OR266121	141	1-21	Yes	*Dufourea novaeangliae*	KZC13557.1	121	3e-56	66.94%
*MsauOBP13*	OR266122	137	1-29	Yes	*Osmia cornuta*	AGI05203.1	137	4e-17	43.51%
*MsauOBP14*	OR266123	137	1-31	Yes	*Osmia cornuta*	AGI05203.1	137	6e-10	41.32%
*MsauOBP15*	OR266124	167	1-24	Yes	*Apis florea*	XP_003690403.1	211	4e-59	68.75%
*MsauOBP16*	OR266125	134	1-17	Yes	*Megachile rotundata*	XP_012153429.1	135	2e-44	61.19%
*MsauOBP17*	OR266126	137	1-19	Yes	*Bombus impatiens*	XP_012240863.1	136	5e-37	51.28%
*MsauOBP18*	OR266127	196	1-24	Yes	*Apis florea*	XP_003690403.1	211	5e-59	68.75%
*MsauOBP19*	OR266128	139	1-19	Yes	*Osmia cornuta*	AGI05204.1	122	5e-29	48.39%
*MsauOBP20*	OR266129	138	1-19	Yes	*Osmia cornuta*	AGI05204.1	122	2e-32	49.59%
*MsauOBP21*	OR266130	143	1-19	Yes	*Osmia cornuta*	AGI05200.1	143	4e-68	65.73%

^a^indicates the corresponding gene has been uploaded by another researcher. *MsauOBP6* has the identical protein sequence with *MsauOBP7* and it has been removed from this paper

**Fig. 4:**
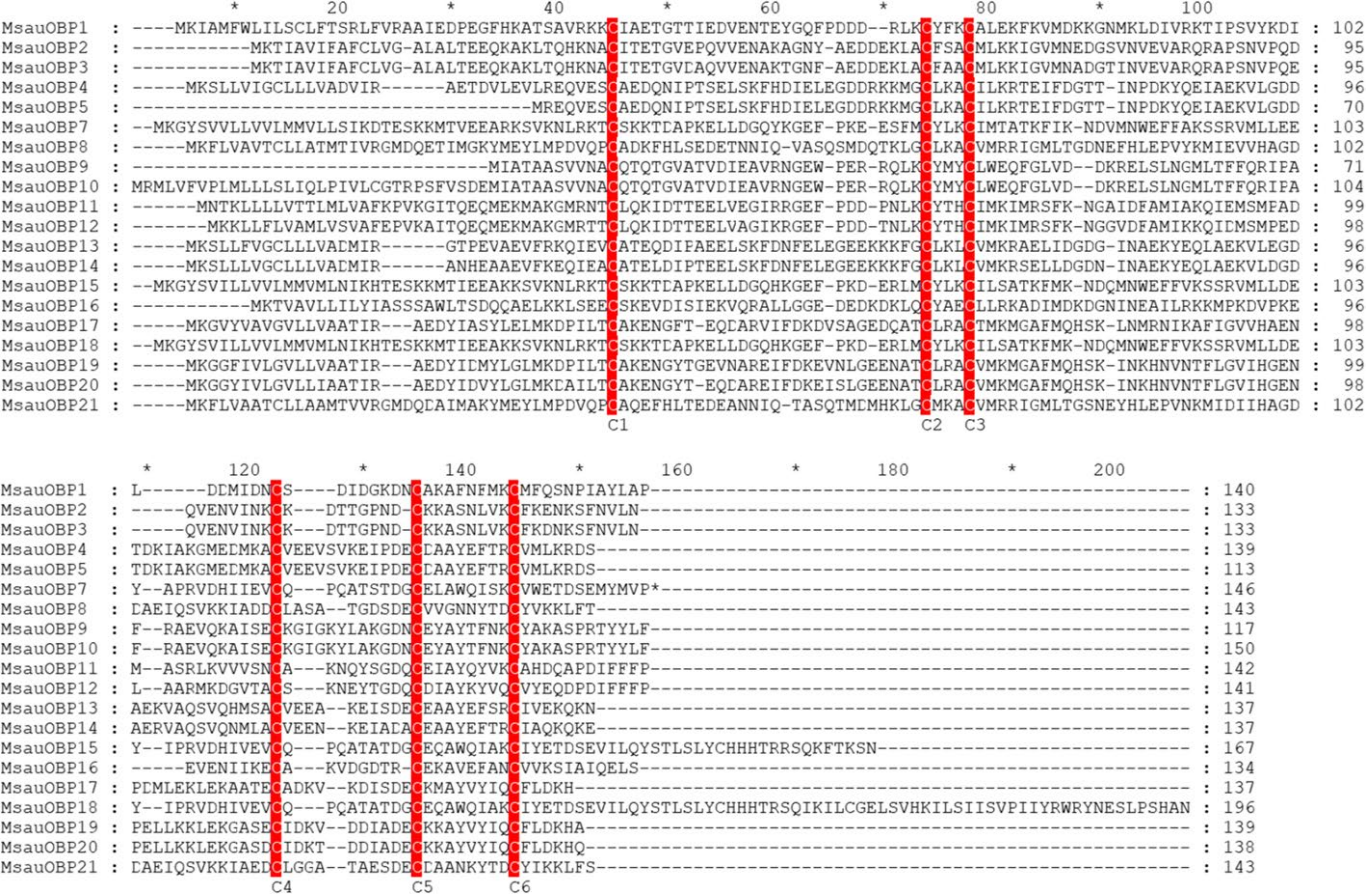
Multiple-sequence alignment of 20 odorant-binding proteins in *M. saussurei* antenna. Red colour represents the six highly-conserved cysteines

**Fig. 5:**
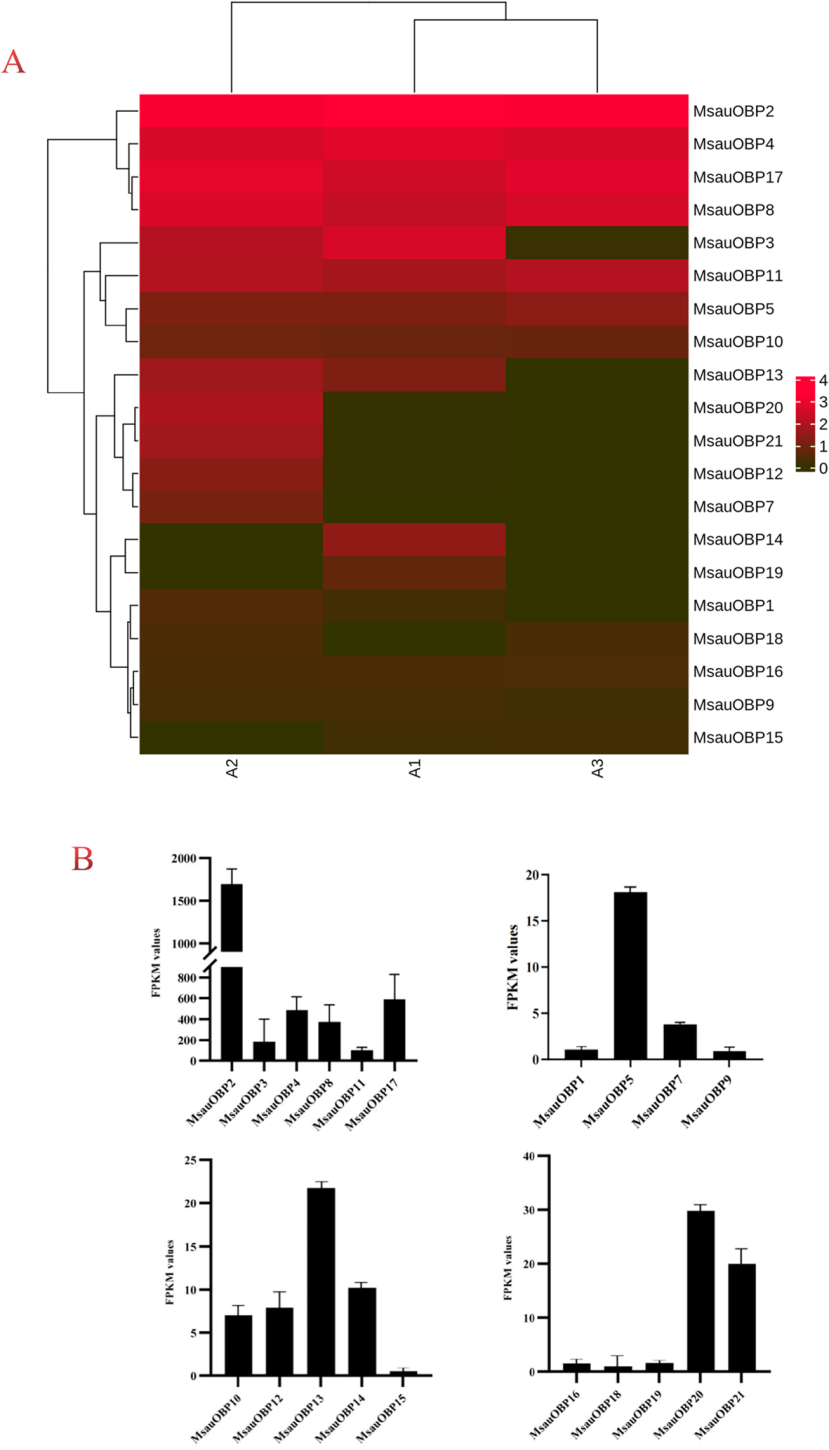
**A** Expression levels of 20 putative odorant-binding proteins from *M. saussurei* antenna (normalized by log_10_(FPKM+1)) in three samples (A1, A2, A3). **B** The actual FPKM values of *M. saussurei* OBPs

Multiple sequence alignment results indicated all putative OBPs displayed highly conserved amino acid sequence structure with six cysteine residues, which belonged to the Classic OBPs subfamily (Fig. 4), while other types of OBPs like Minus-C, Plus-C, Dimer, or Atypical OBPs were not found. The motif structure of MsauOBPs is (C_1_-X_26-28_-C_2_-X_3_-C_3_-X_37-43_-C_4_-X_8-12_-C_5_-X_8_-C_6_), where X*n* stands for any *n* amino acids [9]. The expression level indicated MsauOBP2, 3, 4, 8, 11, and 17 were highly enriched in the *M. saussurei* antenna (Fig. 5B) and the relative expression level of 20 putative odorant-binding proteins in three biological samples of *M. saussure*i antenna was displayed in Fig. 5A.

**Fig. 6:**
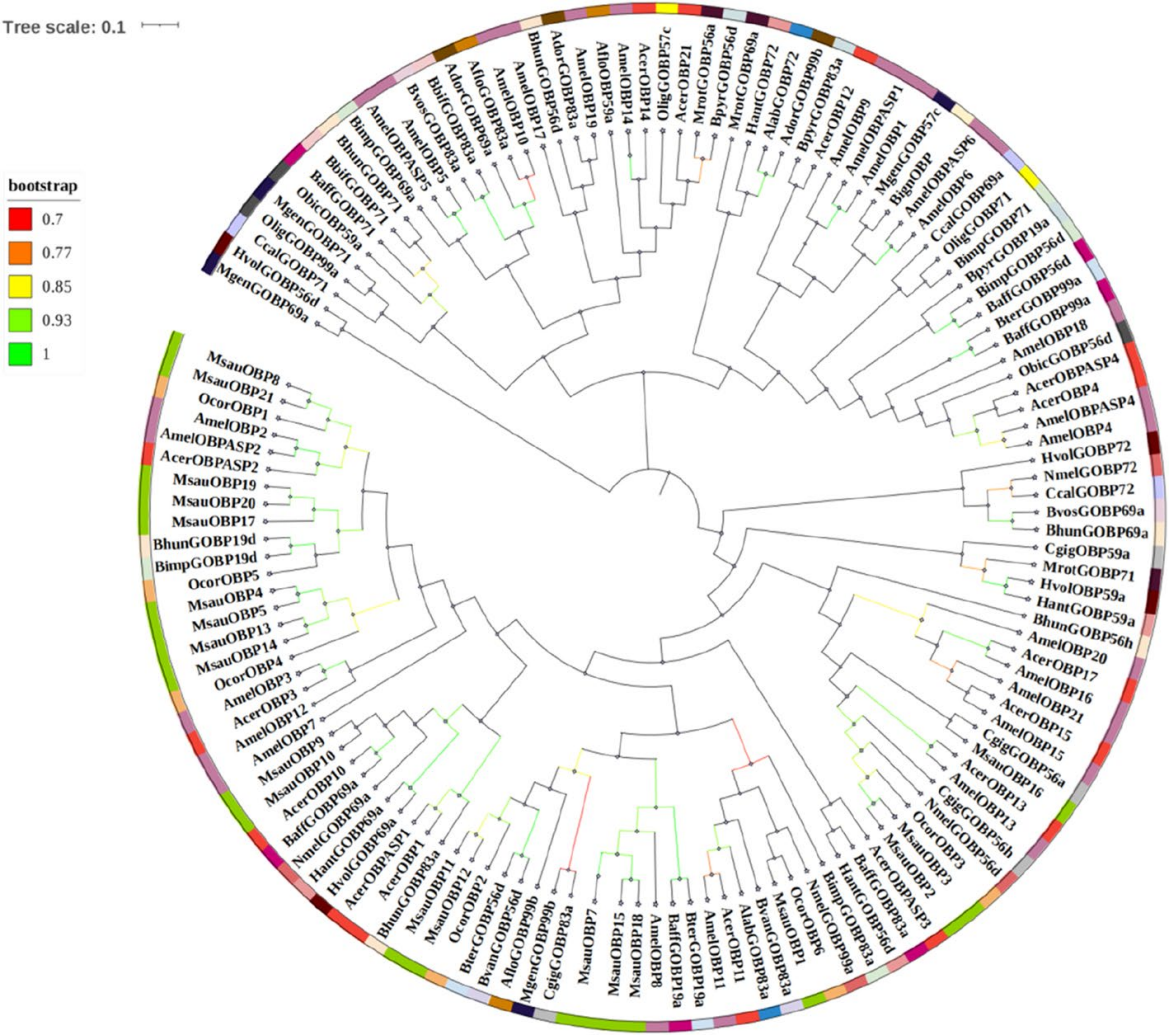
Phylogenetic tree (bootstrap values ≥0.7 were shown) between MsauOBPs and OBPs from other 24 species. Acer: *Apis cerana*; Ador: *Apis dorsata*; Aflo: *Apis florea*; Alab: *Apis laboriosa*; Amel: *Apis mellifera*; Baff: *Bombus affinis*; Bbif: *Bombus bifarius*; Bhun: *Bombus huntii*; Bign: *Bombus ignitus*; Bimp: *Bombus impatiens*; Bpyr: *Bombus pyrosoma*; Bter: *Bombus terrestris*; Bvan: *Bombus vancouverensis*; Bvos: *Bombus vosnesenskii*; Ccal: *Ceratina calcarata*; Cgig: *Colletes gigas*; Hant: *Hylaeus anthracinus*; Hvol: *Hylaeus volcanicus*; Mrot: *Megachile rotundat*a; Mgen: *Megalopta genalis*; Nmel: *Nomia melanderi*; Ocor: *Osmia cornuta*; Olig: *Osmia lignaria*; Obic: *Osmia bicornis*. Green strips represent MsauOBPs. The specific OBP and corresponding accession number were listed in Table S1

To compare the homologous relationship of MsauOBPs with other species, the phylogenetic trees between the identified 20 putative *M. saussurei* OBPs and 119 known OBP protein sequences from other 24 species in Hymenoptera were then constructed (Fig. 6). The tree was divided into three branches, and all MsauOBPs were grouped in the same one. The putative MsauOBPs were most homologous to OcorOBPs of *Osmia cornuta*, among which MsauOBP8 and MsauOBP21 were clustered together with OcorOBP1; MsauOBP17, 19 and 20 were closed to OcorOBP5; MsauOBP4, 5, 13 and 14 were grouped with OcorOBP4; MsauOBP11 and MsauOBP12 were clustered with OcorOBP4; MsauOBP1 was grouped with OcorOBP6; MsauOBP2 and MsauOBP3 were grouped with OcorOBP3. While MsauOBP9 and MsauOBP10 were closed to AcerOBP10 of *Apis cerana*, MsauOBP7, 15, and 18 were closed to AmelOBP8 of *Apis mellifera*, MsauOBP16 was grouped with CgigGOBP56a of *Colletes gigas*.

### Expression analysis of MsauOBPs based on quantitative real-time PCR

**Fig. 7:**
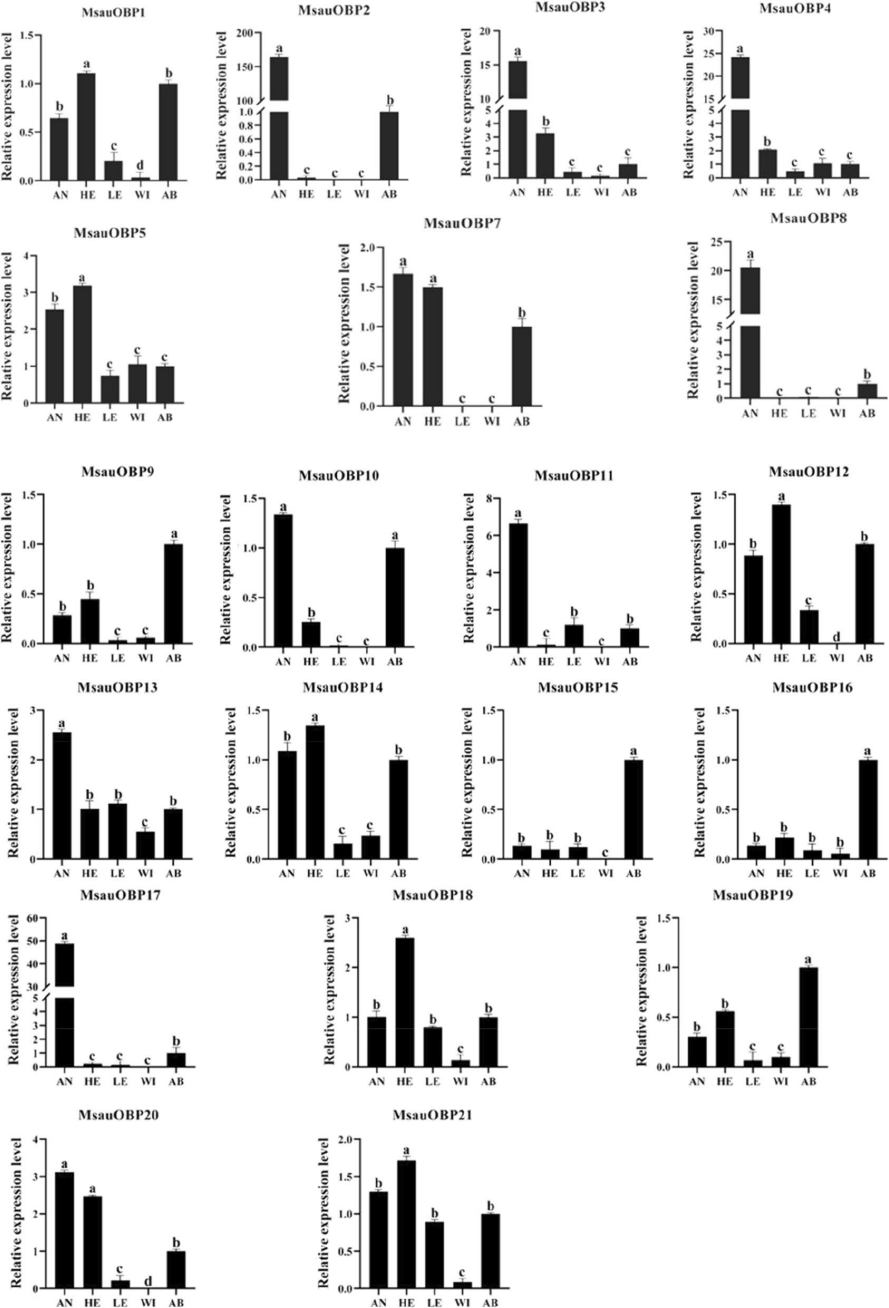
Relative expression levels (mean value ± SD) of *M. saussurei* OBPs in different tissues based on quantitative real-time PCR. Different lowercase letters indicate significant differences (one-way ANOVA followed by Tukey’s test, *p* < 0.05). AN, antennae; HE, head; LE, legs; WI, wing; AB, abdomen. The relative expression level of OBPs in *M. saussurei* abdomen was set to one

To further understand the expression level of 20 putative MsauOBPs, the quantitative real-time PCR experiment was conducted in different tissues. Results showed all MsauOBPs were differentially expressed in antennae and other body parts, and the expression variations were significant (Fig. 7). 10 out of 20 OBPs, including MsauOBP2, 3, 4, 7, 8, 10, 11, 13, 17, and 20, were highly expressed in antennae. While MsauOBP1, 5, 12, 14, 18, and 21 were highly expressed in heads. Only a small number of OBPs were expressed in legs and wings, among which some were even too low to be detected. MsauOBP9, 15, 16, and 19 had a lower expression in all tissues. MsauOBP2, MsauOBP8 and MsauOBP17 had a higher expression in antennae but extremely low expression levels in other body parts, implying their potential olfactory functions.

### Homologous modeling and molecular docking

**Fig. 8:**
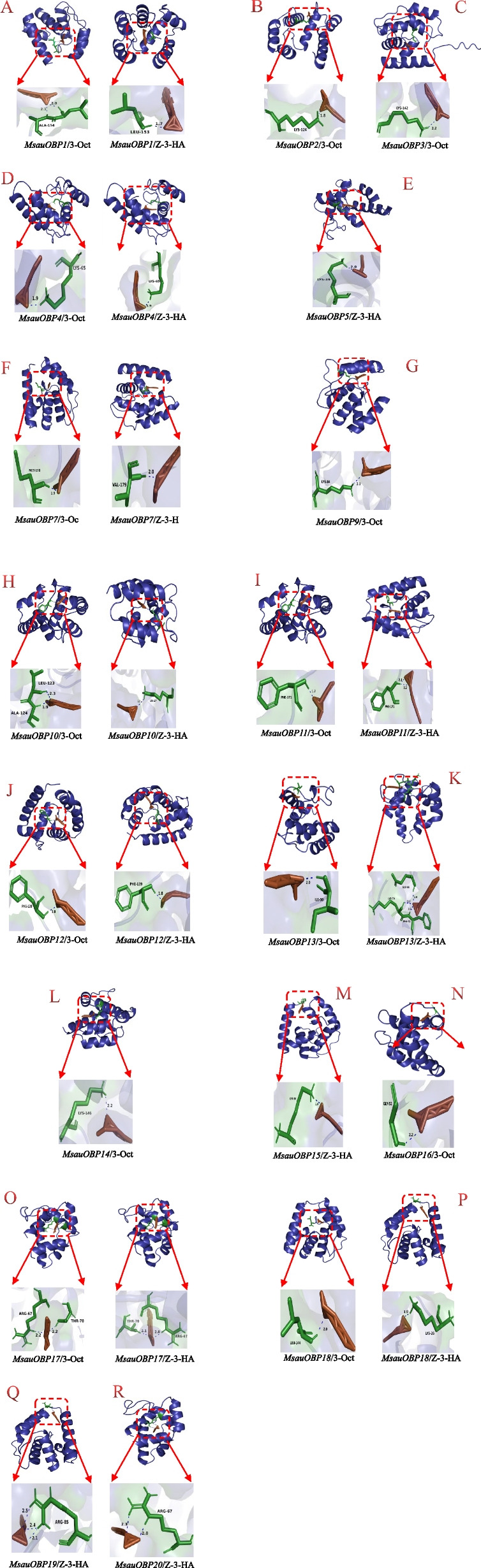
Three-dimensional structures of interaction between M. saussurei and alfalfa flower volatile 3-Octanone and (Z)-3-hexenyl acetate based on molecular docking. **A** Molecular docking simulation of *MsauOBP1*/3-Oct, Z-3-HA; **B**,** C** Molecular docking simulation of *MsauOBP2, MsauOBP3*/3-Oct; **D** Molecular docking simulation of *MsauOBP4*/3-Oct, Z-3-HA; **E** Molecular docking simulation of *MsauOBP5*/Z-3-HA; **F** Molecular docking simulation of *MsauOBP7*/3-Oct, Z-3-HA; **G** Molecular docking simulation of *MsauOBP9*/3-Oct; **H-K** *MsauOBP10-MsauOBP13*/3-Oct, Z-3-HA; **L** Molecular docking simulation of *MsauOBP14*/3-Oct; **M** Molecular docking simulation of *MsauOBP15*/Z-3-HA; **N** Molecular docking simulation of *MsauOBP16*/3-Oct; **O**, **P** Molecular docking simulation of *MsauOBP17, MsauOBP18*/3-Oct, Z-3-HA; **Q** Molecular docking simulation of *MsauOBP19/*Z-3-HA; **R** Molecular docking simulation of *MsauOBP20/*Z-3-HA

Molecular docking results indicated the residues engaged in hydrogen bond (H-bond) formation varied significantly among MsauOBPs. In the docking results of MsauOBPs and 3-Octanone, LYS and LEU were the most frequently appeared residues in forming the H-bond (Table 2). All MsauOBPs successfully formed H-bond with 3-Octanone except for *MsauOBP15*. However, the binding sites of *MsauOBP5*, *MsauOBP8*, *MsauOBP19, MsauOBP20,* and *MsauOBP21* were out of the potential domain of hydrophobic cavity. The *MsauOBP1*, *MsauOBP10*, and *MsauOBP17* possessed two active binding sites, while the others only only had one (Fig. 8). The MsauOBP4 showed the best docking result with a mean binding energy of -20.84 kJ/mol. Similar to 3-Octanone, LYS was also repeatedly used in the docking of MsauOBP-(Z)-3-hexenyl acetate. Four OBPs, *MsauOBP2*, *MsauOBP3*, *MsauOBP9*, and *MsauOBP16*, showed no H-bond formation with (Z)-3-hexenyl acetate, which was greater than the number of OBP when docking with 3-Octanone (Table 2). The binding sites of *MsauOBP8*, *MsauOBP14*, and *MsauOBP21* were out of the potential domain of hydrophobic cavity.Two active forming sites were detected in *MsauOBP14* and 17, while three were found in *MsauOBP13* with the lowest binding energy of -24.02 kJ/mol (Table 2, Fig. 8). Overall, more MsauOBPs tended to combine with 3-Octanone rather than (Z)-3-hexenyl acetate. However, among the MsauOBPs that could form H-bonds with both ligands, the mean binding energy in the docking of MsauOBP-(Z)-3-hexenyl acetate was generally lower than that of MsauOBP-3-Octanone.

**Table 2:** Molecular docking results of MsauOBPs with 3-Octanone and (Z)-3-hexenyl acetate

Gene	3-Octanone		(Z)-3-hexenyl acetate	
	Mean Binding Energy (kJ/mol)	Residues Forming H-Bond	Mean Binding Energy (kJ/mol)	Residues Forming H-Bond
*MsauOBP1*	-15.48	LEU153, ALA154	-16.86	LEU153
*MsauOBP2*	-15.02	LYS124	-15.15	
*MsauOBP3*	-17.61	LYS142	-15.90	
*MsauOBP4*	-20.84	LYS65	-21.00	LYS65
*MsauOBP5*			-21.55	LYS39
*MsauOBP7*	-16.61	MET178	-19.25	VAL179
*MsauOBP8*				
*MsauOBP9*	-15.77	LYS84	-20.29	
*MsauOBP10*	-16.90	LEU123, ALA124	-18.41	LYS117
*MsauOBP11*	-17.66	PHE171	-20.25	PHE171
*MsauOBP12*	-13.31	PHE139	-21.67	PHE139
*MsauOBP13*	-17.11	ILE50	-24.02	GLU66, LYS70, PHE71
*MsauOBP14*	-15.69	LYS146		
*MsauOBP15*	-15.86		-20.54	LYS26
*MsauOBP16*	-17.11	GLY52	-15.73	
*MsauOBP17*	-16.02	ARG67, THR70	-19.92	ARG67, THR70
*MsauOBP18*	-16.07	LEU144	-20.25	LYS26
*MsauOBP19*			-16.69	ARG85
*MsauOBP20*			-14.48	ARG67
*MsauOBP21*				

Those without both binding energy and residues forming H-Bond indicate the binding sites are out of the domain of hydrophobic cavity

## Discussion

Antennae are one of the most crucial sensory organs in insects, in which the chemosensory system contains multiple olfactory genes receiving chemical odors to mediate insect behaviors [35, 36]. Especially the odorant-binding proteins (OBPs), which were thought to be the first step in molecular recognition and the transport of semiochemicals [9]. To date, OBPs and their potential functions have been well-studied in many species, including *Nasonia vitripennis* [37], *Leptinotarsa decemlineata* [38], *Cybister japonicus* [39], *Bemisia tabaci* [40] (Zeng et al., 2019), *Conogethes pinicolalis* [41] and *Meteorus pulchricornis* [26] etc. *M. saussurei* was found to be an efficient pollinator of alfalfa in Northwestern China, and not much research was conducted against this species. To further understand the chemosensory information of *M. saussurei*, we conducted the antennal transcriptome to screen and identify the putative OBPs that might be involved in recognizing external odors and regulating their pollinating behaviors.

Based on our transcriptome results, 20 putative OBPs of *M. saussurei* were discovered, which contained 6 highly-conserved cysteines. The number was less than that of *Nasonia vitripennis* [37] (90 OBPs), similar to *A. mellifera* (21 OBPs) [42] and *Cotesia vestalis* (20 OBPs) [28], but more than *M. rotundata* (7 OBPs) [6], respectively. All putative OBPs belonged to the subgroup of Classic OBPs (Fig. 2) and encoded 143 amino acids on average, which was in a reasonable range compared with other species [28, 43, 44]. It has been pointed out that the number of OBPs could vary significantly across species [45]. In species where genome data has been published, for instance, Orthoptera, *Locusta migratoria* (22 OBPs); Hemiptera, *Acyrthosiphon pisum* (16 OBPs); Coleoptera, *Tribolium castaneum* (50 OBPs); Lepidoptera, *Bombyx mori* (45 OBPs); Hymenoptera, *Apis mellifera* (21 OBPs); *M. rotundata* (7 OBPs); *Nasonia vitripennis* (90 OBPs); Diptera, *Drosophila melanogaster* (52 OBPs) [6, 10]. It was hypothesized the diverse OBPs were probably related to the various semiochemicals in our environment [46], which formed the basis that different OBPs perform disparate functions. Besides, the real number of putative OBPs in *M. saussurei* could be more than 20, because a few OBPs were specifically expressed in other tissues of insects, such as gut [47], genital [48], legs and wings [49]. It’s highly possible that other OBPs were not included in our results, which might be one reason that we were not able to identify other types of OBPs.

The phylogenetic tree was often used to compare the homology relationship between different species. In this research, we collected 119 OBPs that were previously uploaded to GenBank from 24 species in Hymenoptera and constructed a phylogenetic tree with 20 predicated MsauOBPs. Results showed all MsauOBPs were clustered into the same branch, indicating there was certain homology existed. However, some differentiation could also be noticed, because not all sequences were grouped in the same clade. For instance, *MsauOBP1* and *MsauOBP16* were respectively clustered with other species, while other MsauOBPs were grouped in pairs or multi numbers (Fig. 4), which was similar to the phylogenetic results in other studies [28, 41, 44]. This divergence was probably due to the complex functions that different MsauOBPs performed. Previous studies even reported OBPs were extremely divergent in their sequences and identical amino acids between members of the same species, as well as between species, might be even lower than 10% [6]. Furthermore, our results also found that *Osmia cornuta* was the most homologous-closed species to *M. saussurei* in OBP sequences, which also belongs to the Megachilidae but in a different genus (Osmia), implying OBPs of these two species may share similar physiological functions.

Expression analysis with quantitative real-time PCR method indicated most putative MsauOBPs were highly expressed in antennae. Although a certain degree of expression could be seen in other tissues, such as *MsauOBP5*, *MsauOBP13*, *MsauOBP18,* and *MsauOBP21* (Fig. 5), more MsauOBPs were presented as antennal-specific expression patterns, especially the *MsauOBP2*, *MsauOBP8,* and *MsauOBP17*, where the maximum differential expression occurred between antennae and other body parts, indicating the olfactory function these OBPs may possess. Similar results have been recorded in *C. vestalis,* in which the *CvesOBP7*, *CvesOBP8*, *CvesOBP13*, *CvesOBP17*, *CvesOBP18,* and *CvesOBP19* were specifically enriched in female antennae, while *CvesOBP9,* and *CvesOBP10* were significantly expressed in bodies [28]. In honey bees, *AmelOBP9* and *AmelOBP10* were reported to be highly expressed in non-olfactory tissues including brains, ovaries, and even eggs except many other antennal-specific enriched OBPs [42]. Besides, the SinvOBP10 of *Solenopsis invicta*, an imported fire ant, was also highly expressed in their brains at the pupal stage [50].

It has been reported the expression variation in different tissues probably corresponded to diverse physiological functions [51, 52]. For instance, the antennal-specific expressed *AcerOBP1* can bind to the main components of the queen pheromones 9-ODA and 9-HDA (9-hydroxy-2(E)-decenoic acid) [53]. The leg-specific expressed AlinOBP11 of *Adelphocoris lineolatus* had important gustatory functions [54]*.* In some Lepidoptera insects, OBPs enriched in bodies may have the function of helping release the semiochemicals [41]. In this study, antennal-specific expressed OBPs, such as MsauOBP2, 3, 4, 8, 11, and 17, were highly possible to possess the olfactory function, which was similar to the fig wasp *Wiebesia pumilae,*, where this creature located its host *Ficus pumila* mainly through *WpumOBP2* binding the decanal emitted by *F. pumila* [55]. Furthermore, the *O.lotOBP6* of *Odontothrips loti* could strongly bind to p-Menth-8-en-2-one emitted by its host *Medicago sativa* and was the most crucial OBP in host-seeking [15]. Consequently, it’s reasonable to hypothesize that *M. saussurei* locate *M. sativa* through these highly expressed OBPs binding the single or multiple volatiles emitted by *M. sativa* to complete their feeding and pollination.

The interaction of MsauOBPs and two alfalfa flower volatiles 3-octanone and (Z)-3-hexenyl acetate was simulated by molecular docking method. Results showed most MsauOBPs could successfully bind with two ligands. It has been confirmed that the lower the binding energy, the better the binding effect [32]. In this study, *MsauOBP4* showed the minimum value of binding energy when docking with 3-Octanone, while *MsauOBP13* presented the lowest binding energy when docking with (Z)-3-hexenyl acetate. This implied *MsauOBP4* and *MsauOBP13* may play a crucial role in recognizing these two volatiles and may also contribute to the host location during the pollination process. Although more MsauOBPs tended to bind with 3-octanone, the mean binding energy of (Z)-3-hexenyl acetate was generally much lower, indicating that the combination between MsauOBPs and (Z)-3-hexenyl acetate was much more stable. Results also found the amino acid lysine appeared most frequently in docking simulations, which was also a momentous amino acid in other soluble olfactory proteins such as *FoccOBP6* of *Frankliniella occidentalis* [8], *OBP3* of *Nilaparvata lugens* [56] and *MsepCSP14* of *Mythimna separata* [57]. It was found hydrophilic amino acids are more likely to form hydrogen bonds with ligands [58]. For instance, asparagine and serine in Hymenoptera [59], arginine, threonine, and aspartic acid in Lepidoptera [60, 61], glutamine in Hemiptera [62]. This was consistent with our result, in which lysine was also one of the hydrophilic amino acids.

## Conclusions

In this study, we identified the OBPs, and conducted the phylogenetic and expression analysis. The interaction between two alfalfa flower volatiles and MsauOBPs was also simulated. Most OBPs were homologous while a certain degree of differences also existed. Six OBPs (MsauOBP2, 3, 4, 8, 11, and 17) mostly enriched in antennae were possibly involved in the olfactory functions. MsauOBP4 might be the key protein in recognizing 3-Octanone, while MsauOBP13 might be the key protein in binding (Z)-3-hexenyl acetate. These two proteins might contribute to the alfalfa-locating during the pollination process. The relevant results may help determine the highly specific and effective attractants for *M. saussurei* in alfalfa pollination and reveal the molecular mechanism of odor-evoked pollinating behavior between these two species. Further studies of these highly expressed OBPs using multi-methods are quite necessary, such as fluorescence binding assay, RNAi technique, and corresponding behavioral experiments, etc. Because these methods have been frequently used for the functional prediction and verification of insect OBPs. The relevant results may help determine the highly specific and effective attractants for *M. saussurei* in alfalfa pollination and reveal the molecular mechanism of odor-evoked pollinating behavior between these two species.

## Methods

### Antenna sample collection

The *M. saussurei* adults were captured in a blooming alfalfa field in the Yumen area (40^◦^45´N, 97^◦^36´E), Gansu province, China, in July 2022. To attract *M. saussurei*, the artificial foam nest (polystyrene bee board) was placed near the edges of the alfalfa field with the openings of the artificial nests facing the alfalfa field in a southeast direction [63, 64]. The size of artificial nests was maintained as instructed by Pitts-Singer and Bosch [65]. After *M. saussurei* was nested in these artificial nests, the emergence status and sex information of the adults were recorded every day. We carefully dissected the antennae from female *M. saussurei* in the laboratory and placed them in 1.5mL centrifugal tubes containing the RNA later buffer solution (Invitrogen, Carlsbad, CA, USA) [18]. The tubes were preserved at -80℃ until RNA extraction.

### RNA extraction and transcriptome sequencing

Fifty pairs of antennae from *M. saussurei* adult females were used for total RNA extraction using TRIzol Reagent (Invitrogen, Waltham, MA, USA) following the manufacturer’s standard protocols (50 pairs antennae formed one sample, three samples (A1, A2, and A3) were set in total). The concentration and quality of RNA were verified using Fragment Analyzer 5200 (Agilent Technologies, Palo Alto, Canada). The cDNA library construction and transcriptome sequencing were performed on the DNBSEQ-500 platform at Wuhan BGI Technology (Wuhan, China) and a detailed flowchart was displayed in Fig. S1.

### De novo assembly and functional annotation

To ensure the data reliability, we obtained clean reads from raw reads by filtering and deleting those reads of low quality, containing adapters and over 5% unknown bases. The clean reads were then assembled with Trinity v2.0.6 (https://github.com/trinityrnaseq/trinityrnaseq/wiki) using default parameters [66]. Then the unigenes from the three samples were pooled together to form the “all-unigene” by clustering reads and removing redundancy with the TGI Clustering Tool (TGICL) [67]. The quality of the assembled transcripts (unigenes) was thereafter evaluated using the BUSCO (Benchmarking Universal Single-Copy Orthologs) (https://busco.ezlab.org/), and the integrity of the transcriptome assembly was illustrated by comparison with conserved genes.

The coding sequence (CDS) in unigenes was identified using TransDecoder software by first extracting the longest open reading frame, and then Blast comparison against the Pfam protein homologous sequences in the SwissProt database and Hmmscan search to predict the coding regions. The unigenes were annotated against seven publicly accessed databases, the Kyoto Encyclopedia of Genes and Genomes (KEGG), the Gene Ontology (GO), the Non-redundant Protein Sequence Database (NR), Nucleotide Sequence Database (NT), the Protein Families Database (Pfam), Swiss-prot protein sequence database (Swiss-prot) and clusters of orthologous groups for eukaryotic complete genomes (KOG) with a threshold E-value < 1e^-5^. The expression level of each unigene was calculated by RSEM software (RNA-Seq by Expectation Maximization) with default parameters and presented as FPKM (fragments per kilobase of transcript per million mapped fragments) values.

### Identification of odorant-binding protein genes and phylogenetic analysis

Candidate unigenes encoding putative odorant-binding proteins (OBPs) were selected from the assembly results. They were manually checked by performing a BLASTx search against the NR database with a threshold E-value < 1e^-5^ [68]. The open reading frame (ORF) of candidate OBP genes was predicted by NCBI ORF Finder (https://www.ncbi.nlm.nih.gov/orffinder). The N-terminal signal peptides were predicted by Signal P4.0 (http://www.cbs.dtu.dk/services/SignalP/).

We applied multiple amino acid sequence alignment with MUSCLE and constructed phylogenetic trees of putative OBP genes using the neighbor-joining (NJ) method with default parameters in MEGA v11.0 software. The reliability of the tree structure and node support was assessed using a bootstrap method with 1000 replicates and the phylogenetic tree was visualized in the Interactive Tree of Life (iTOL) (https://itol.embl.de/). Sequences of OBP genes from other bees were searched and selected from NCBI and used in the phylogenetic tree construction (Table S1). We finally aligned putative OBPs using GenDoc software and determined the type of putative OBPs.

### Expression analysis by quantitative real-time PCR

After we identified the OBPs from the antennal transcriptome, we verified their expression levels in different tissues of *M. saussurei* using the quantitative real-time PCR method (RT-qPCR). Antenna, heads, legs, wings, and abdomen from 20 individuals were respectively collected and pooled together as one sample. Total RNA was extracted with TRIzol reagent (Invitrogen) and the cDNA was synthesized using the PrimeScript RT Reagent Kit with gDNA Eraser (TaKaRa, Shiga, Japan). The total volume of the PCR reaction system was 25μl, which contains 12.5 µl of SYBR Premix Ex Taq^TM^, 0.5 µl of forward primer, 0.5 µl of reverse primer, 2 μl of sample cDNA and 8.5 μl of double-distilled H_2_O. This PCR system was performed under the conditions of 95℃ for 30 s; 40 cycles of 95℃ for 5 s and 60℃ for 30 s; 65℃ to 95℃ in increments of 0.5℃ for 5 s. Negative controls with ddH_2_O were included. Gene-specific primers (Table S2) were designed using the Primer 3.0 plus server in NCBI. Nuclear β-actin was used as the internal reference gene and abdomen samples were used as the control group. Three biological replicates and three technical replicates were applied for each experiment.

The relative expression level of OBP genes was normalized using the comparative 2^−∆∆Ct^ method [69]. One-way ANOVA analysis was applied to compare the expression levels between tissues, followed by Tukey’s post hoc comparison test for the significant differences. The data analysis and plot-making were both conducted using GraphPad Prism 9.0 software.

### Homologous modeling and molecular docking

The online platform SWISS-MODEL (https://swissmodel.expasy.org) was used to predict the three-dimensional structure of all MsauOBPs. Models with similarity >30% were selected as reference templates. The PROCHECK program [70] was used to assess the generated MsauOBP models. 3-Octanone and (Z)-3-hexenyl acetate are two main components of alfalfa flower volatiles with relatively high content [71–74]. Ligand molecules were obtained from the PubChem database (https://pubchem.ncbi.nlm.nih.gov). The Autodock 4.2.6 and AutoDock Tools 1.5.7 with default parameters were used to conduct the molecular docking between MsauOBPs and two ligands. The docking results were visualized by PYMOL software.

### Supplementary Information

**Additional file 1: Figure S1.** Flow chart of mRNA library construction. **Figure S2.** The distribution in sequence size of all unigenes. **Figure S3.** The assembly evaluation based on BUSCO. Complete: Sequences that matched to the records of the BUSCO database; F(fragmented): Partial sequences that matched to the records of the BUSCO database; D(duplicate): Multiple genes matched to one record of the BUSCO database; M(missing): Sequences that were filtered out. **Table S1.** OBP genes information of other species in phylogenetic analysis. **Table S2.** Gene-specific primers used for quantitative real-time PCR. **Table S3.** Quality statistics of filtered Reads in transcriptome sequencing. **Table S4.** The quality indicators of unigenes after Denovo assembly. **Table S5.** Functional annotation results of unigenes.

## Data Availability

All data support this research is included in this article and supplementary file. The original reads of transcriptome sequencing from this study were uploaded to NCBI Sequence Read Archive with accession number PRJNA977226. The sequences of 20 MsauOBPs were also submitted to Genbank with accession number OR266110-OR266114, OR266116-OR266130. The internal reference gene, Nuclear β-actin, was obtained from transcriptome sequencing data with Genbank accession number OR405375.

## Abbreviations

OBPs: Odorant binding proteins
MsauOBPs: *Megachile saussurei* odorant binding proteins
ORs: odorant receptors
CSPs: chemosensory proteins
IRs: ionotropic receptors
SNMPs: sensory neuron membrane proteins
ODEs: odorant-degrading enzymes
TGICL: TIGR Gene Indices clustering tools
BUSCO: Benchmarking Universal Single-Copy Orthologs
CDS: coding sequence
ORFs: Open reading frame
KEGG: Kyoto Encyclopedia of Genes and Genomes
GO: Gene ontology
NR: Non-redundant protein sequence database
NT: Nucleotide Sequence Database
Pfam: Protein families database
Swiss-prot: Swiss-prot protein sequence database
KOG: clusters of orthologous groups for eukaryotic complete genomes
RSEM: RNA-Seq by Expectation Maximization
FPKM: Fragments per kilobase per million reads
RT-qPCR: Fluorescent quantitative real-time PCR
