# Supplementary material for: The antennal transcriptome analysis and characterizations of odorant-binding proteins in Megachile saussurei (Hymenoptera, Megachilidae)
Source: BMC Genomics. 2023 Dec 15;24:781. doi: 10.1186/s12864-023-09871-8 (PMC10724985; doi:10.1186/s12864-023-09871-8)
Supplement: Supplementary file 1 — Additional file 1: Figure S1. Flow chart of mRNA library construction. Figure S2. The distribution in sequence size of all unigenes. Figure S3. The assembly evaluation based on BUSCO. Complete: Sequences that matched to the records of the BUSCO database; F(fragmented): Partial sequences that matched to the records of the BUSCO database; D(duplicate): Multiple genes matched to one record of the BUSCO database; M(missing): Sequences that were filtered out. Table S1. OBP genes information of other species in phylogenetic analysis. Table S2. Gene-specific primers used for quantitative real-time PCR. Table S3. Quality statistics of filtered Reads in transcriptome sequencing. Table S4. The quality indicators of unigenes after Denovo assembly. Table S5. Functional annotation results of unigenes. [file 12864_2023_9871_MOESM1_ESM.docx]

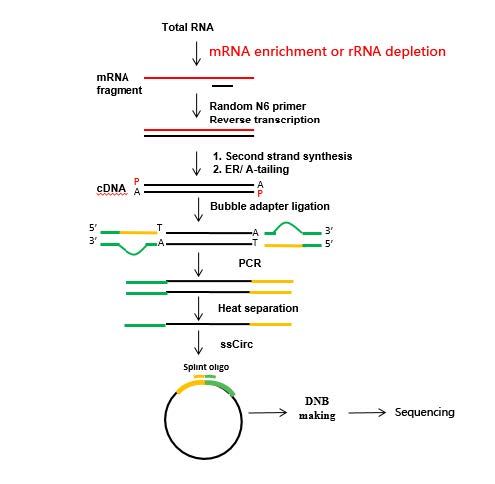


Figure S1 Flow chart of mRNA library construction


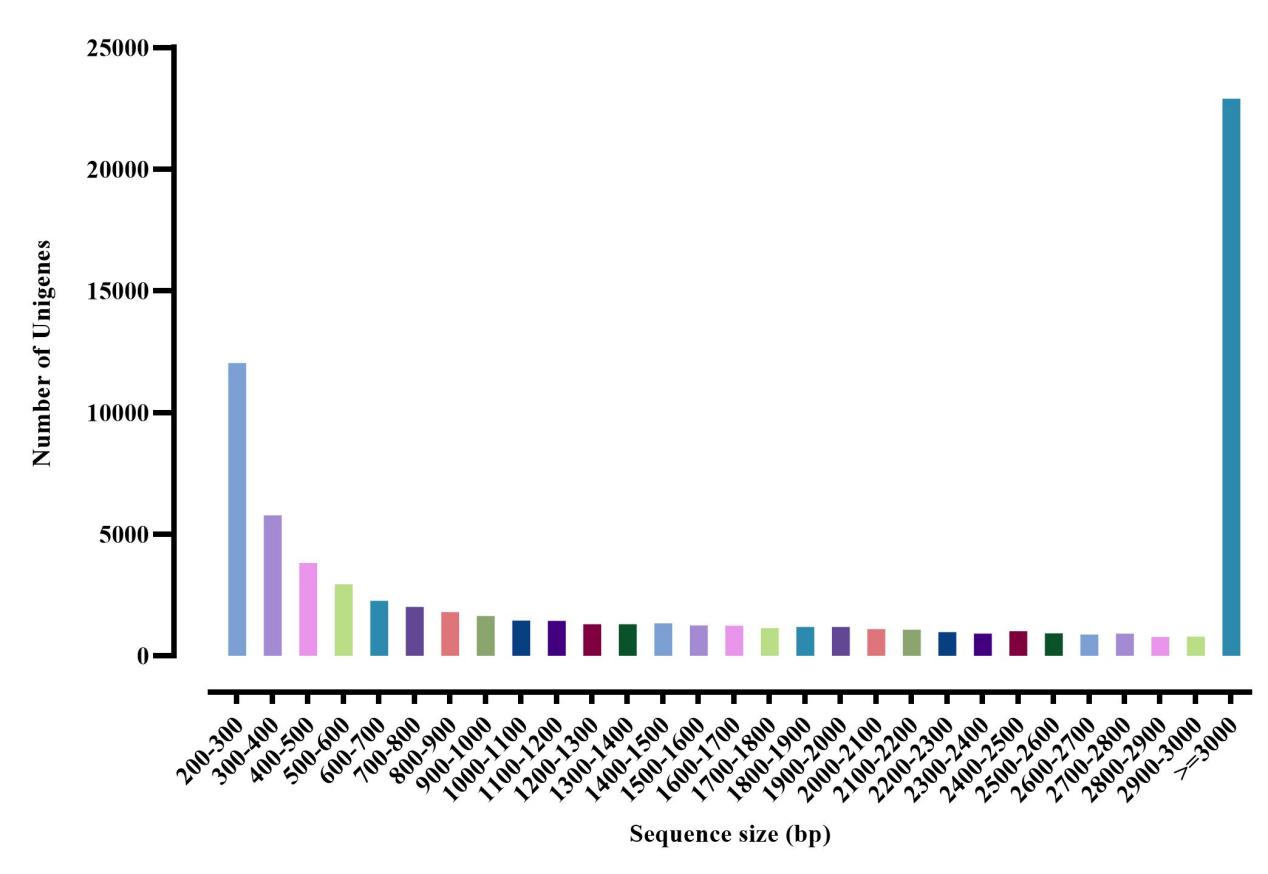


Figure S2 The distribution in sequence size of all unigenes


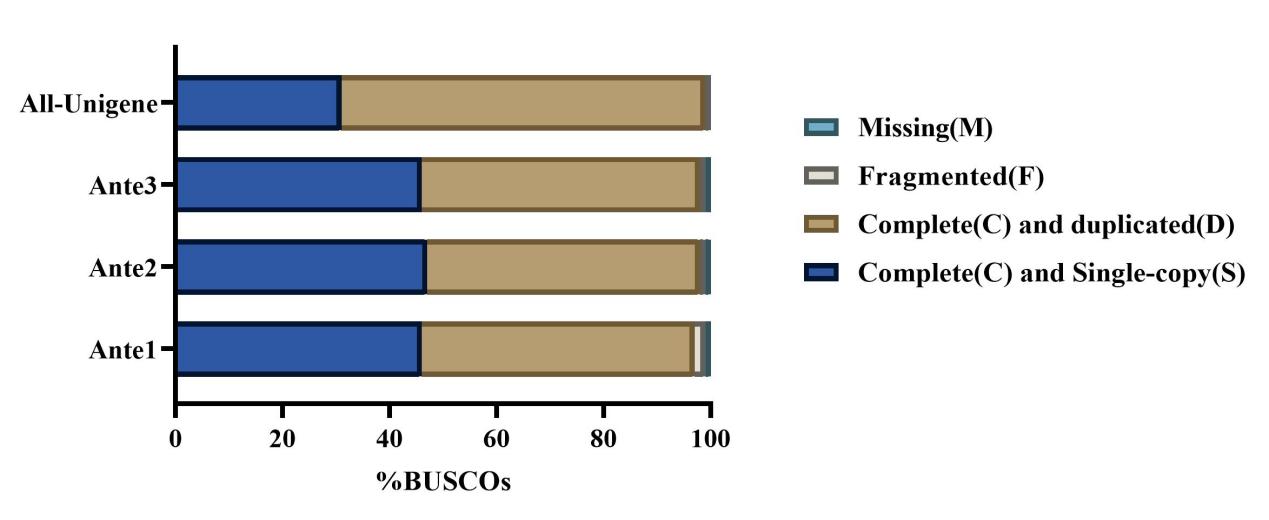


Assembly evaluation (%)

Sample

Figure S3 The assembly evaluation based on BUSCO. Complete: Sequences that matched to the records of the BUSCO database; F(fragmented): Partial sequences that matched to the records of the BUSCO database; D(duplicate): Multiple genes matched to one record of the BUSCO database; M(missing): Sequences that were filtered out.

| Table S1 OBP genes information of other species in phylogenetic analysis | | |
| --- | --- | --- |
| Species | Gene name | Accsession number |
| Apis cerana | OBP21 | KP717063.1 |
| Apis cerana | OBP14 | KP717062.1 |
| Apis cerana | OBP12 | KP717061.1 |
| Apis cerana | OBP10 | KP717060.1 |
| Apis cerana | OBP4 | KP717059.1 |
| Apis cerana | OBP11 | KC818631.1 |
| Apis cerana | OBP1 | JQ003219.1 |
| Apis cerana | OBPASP4 | AY392756.1 |
| Apis cerana | OBPASP1 | DQ449670.1 |
| Apis cerana | OBP15 | KT588076.1 |
| Apis cerana | OBP13 | KT279587.1 |
| Apis cerana | OBP12 | KT279586.1 |
| Apis cerana | OBP14 | KT246482.1 |
| Apis cerana | OBPASP2 | DQ449667.2 |
| Apis cerana | OBP17 | KM591216.1 |
| Apis cerana | OBP3 | KJ026357.1 |
| Apis cerana | OBP1 | JQ003218.1 |
| Apis cerana | OBPASP3 | DQ449669.2 |
| Apis dorsata | GOBP99b | XM_006616876.2 |
| Apis dorsata | GOBP69a | XM_006620386.2 |
| Apis dorsata | GOBP83a | XM_006620384.2 |
| Apis florea | OBP59a | XM_031915689.1 |
| Apis florea | GOBP83a | XM_003692743.3 |
| Apis florea | GOBP99b | XM_003690364.3 |
| Apis laboriosa | GOBP83a | XM_043931160.1 |
| Apis laboriosa | GOBP72 | XM_043933813.1 |
| Apis mellifera | OBP2 | NM_001011591.1 |
| Apis mellifera | OBP1 | NM_001011590.1 |
| Apis mellifera | OBP6 | NM_001011593.1 |
| Apis mellifera | OBP14 | NM_001040223.1 |
| Apis mellifera | OBP11 | NM_001040226.1 |
| Apis mellifera | OBP5 | NM_001011588.1 |
| Apis mellifera | OBP4 | NM_001011589.1 |
| Apis mellifera | OBP18 | NM_001040227.1 |
| Apis mellifera | OBP13 | NM_001040224.1 |
| Apis mellifera | OBP19 | NM_001040209.1 |
| Apis mellifera | OBP21 | NM_001040206.1 |
| Apis mellifera | OBP17 | NM_001040207.1 |
| Apis mellifera | OBP16 | NM_001040205.1 |
| Apis mellifera | OBP10 | NM_001040204.1 |
| Apis mellifera | OBP15 | NM_001040208.1 |
| Apis mellifera | OBP9 | NM_001040208.1 |
| Apis mellifera | OBP8 | NM_001171044.1 |
| Apis mellifera | OBP12 | NM_001040229.1 |
| Apis mellifera | OBP3 | NM_001040229.1 |
| Apis mellifera | OBP7 | NM_001040220.1 |
| Apis mellifera | OBP20 | NM_001040222.1 |
| Apis mellifera | OBPASP5 | AF393497.1 |
| Apis mellifera | OBPASP6 | AF393496.1 |
| Apis mellifera | OBPASP4 | AF393495.1 |
| Apis mellifera | OBPASP1 | AF393494.1 |
| Apis mellifera | OBPASP2 | AF393493.1 |
| Bombus affinis | GOBP56d | XM_050731449.1 |
| Bombus affinis | GOBP19a | XM_050731442.1 |
| Bombus affinis | GOBP69a | XM_050744686.1 |
| Bombus affinis | GOBP99a | XM_050744684.1 |
| Bombus affinis | GOBP83a | XM_050738716.1 |
| Bombus affinis | GOBP71 | XM_050734492.1 |
| Bombus bifarius | GOBP71 | XM_033462478.1 |
| Bombus bifarius | GOBP83a | XR_004487910.1 |
| Bombus huntii | GOBP19d | XM_050619298.1 |
| Bombus huntii | GOBP56d | XM_050619286.1 |
| Bombus huntii | GOBP83a | XM_050623474.1 |
| Bombus huntii | GOBP56h | XM_050627600.1 |
| Bombus huntii | GOBP71 | XM_050630685.1 |
| Bombus huntii | GOBP69a | XM_050619658.1 |
| Bombus ignitus | OBP | EU365128.1 |
| Bombus impatiens | GOBP69a | XM_033323674.1 |
| Bombus impatiens | GOBP83a | XR_002947640.2 |
| Bombus impatiens | GOBP71 | XM_012384391.3 |
| Bombus impatiens | GOBP56d | XM_012385366.3 |
| Bombus impatiens | GOBP19d | XM_012385440.3 |
| Bombus pyrosoma | GOBP56d | XM_043746251.1 |
| Bombus pyrosoma | GOBP19a | XM_043734635.1 |
| Bombus pyrosoma | GOBP83a | XM_043736195.1 |
| Bombus terrestris | GOBP56d | XM_003397873.3 |
| Bombus terrestris | GOBP19a | XM_048408952.1 |
| Bombus terrestris | GOBP99a | XM_012312928.3 |
| Bombus vancouverensis | GOBP56d | XM_033338065.1 |
| Bombus vancouverensis | GOBP83a | XR_004464424.1 |
| Bombus vosnesenskii | GOBP69a | XM_033495689.1 |
| Bombus vosnesenskii | GOBP83a | XR_004493530.1 |
| Ceratina calcarata | GOBP72 | XM_018027172.2 |
| Ceratina calcarata | GOBP69a | XM_018036463.2 |
| Ceratina calcarata | GOBP71 | XM_026815082.1 |
| Colletes gigas | GOBP83a | XM_043395596.1 |
| Colletes gigas | OBP59a | XM_043399160.1 |
| Colletes gigas | GOBP56h | XM_043410101.1 |
| Colletes gigas | GOBP56a | XM_043409909.1 |
| Hylaeus anthracinus | GOBP69a | XM_054159358.1 |
| Hylaeus anthracinus | OBP59a | XM_054155665.1 |
| Hylaeus anthracinus | OBP72 | XM_054141043.1 |
| Hylaeus anthracinus | GOBP56d | XM_054159805.1 |
| Hylaeus volcanicus | OBP59a | XM_054130384.1 |
| Hylaeus volcanicus | GOBP69a | XM_054123793.1 |
| Hylaeus volcanicus | GOBP56d | XM_054120876.1 |
| Hylaeus volcanicus | GOBP72 | XM_054116480.1 |
| Megachile rotundata | GOBP56a | XM_003708502.2 |
| Megachile rotundata | GOBP69a | XM_003701405.2 |
| Megachile rotundata | GOBP71 | XM_003705175.2 |
| Megalopta genalis | GOBP71 | XM_033465428.1 |
| Megalopta genalis | GOBP99b | XM_033471915.1 |
| Megalopta genalis | GOBP69a | XM_033467777.1 |
| Megalopta genalis | GOBP57c | XM_033472039.1 |
| Nomia melanderi | GOBP56d | XM_031987225.1 |
| Nomia melanderi | GOBP69a | XM_031992042.1 |
| Nomia melanderi | GOBP99a | XM_031992032.1 |
| Nomia melanderi | GOBP72 | XM_031985122.1 |
| Osmia cornuta | OBP5 | KC464553.1 |
| Osmia cornuta | OBP6 | KC464554.1 |
| Osmia cornuta | OBP4 | KC464552.1 |
| Osmia cornuta | OBP3 | KC464551.1 |
| Osmia cornuta | OBP2 | KC464550.1 |
| Osmia cornuta | OBP1 | KC464549.1 |
| Osmia lignaria | GOBP71 | XM_034321894.1 |
| Osmia lignaria | GOBP57c | XM_034329858.1 |
| Osmia bicornis | GOBP99a | XM_029183888.2 |
| Osmia bicornis | OBP59a | XM_029186310.2 |
| Osmia bicornis | GOBP56d | XM_029191810.2 |

| Table S2 Gene-specific primers used for quantitative real-time PCR | | |
| --- | --- | --- |
| Gene name | Primer | Sequence |
| MsauOBP1 | forward | AAGGACAATTGCGCGAAAGC |
|  | reverse | ATGGCGCGAGGTAAGCAATA |
| MsauOBP2 | forward | ATGAACTGGCCTGCTTCTCC |
|  | reverse | CTTGAGGGACGTTGGATGGA |
| MsauOBP3 | forward | AGACCGGTGTAGACGCCC |
|  | reverse | CGTTCATGACGCCGATTTTC |
| MsauOBP4 | forward | CCCTTTTGGTGATCGGTTGC |
|  | reverse | TCCTCGGCACAGGATTCAAC |
| MsauOBP5 | forward | AGGTTGAATCCTGTGCCGAG |
|  | reverse | ACAAGCCTTCAAGCAACCCA |
| MsauOBP6 | forward | ACATGCAGCAAGAAGACCGA |
|  | reverse | ACTCGTGGTGCGTATTCCTC |
| MsauOBP7 | forward | ACATGCAGCAAGAAGACCGA |
|  | reverse | ACTCGTGGTGCGTATTCCTC |
| MsauOBP8 | forward | ACGATTGTTCGCGGTATGGA |
|  | reverse | CCATCGACTGTGAGGCTACC |
| MsauOBP9 | forward | TGAACGCTTGTCAAACGCAG |
|  | reverse | TGTCGTCGACCAACCCAAAT |
| MsauOBP10 | forward | CCGTTGATGCTCCTCCTCTC |
|  | reverse | GTCTGCGTTTGACAAGCGTT |
| MsauOBP11 | forward | TGGTGCTCTATTCTGTGCGG |
|  | reverse | ATCCGGGAATTCGCCTCTTC |
| MsauOBP12 | forward | AGATGGCGAAAGGAATGCGA |
|  | reverse | TGAGATTCGTGTCGTCTGGG |
| MsauOBP13 | forward | AAGTTGCTCAAAGCGTGCAG |
|  | reverse | CCACGATGCAACGCGAAAAT |
| MsauOBP14 | forward | CGGCTGCTTGAAGTTGTGTG |
|  | reverse | AACTCTTTCCGCATCACCGT |
| MsauOBP15 | forward | ATTCCGCGAGTTGACCACAT |
|  | reverse | TCGTCGTGTATGGTGATGGC |
| MsauOBP16 | forward | ACATCGCAAGTAGTTCGGCT |
|  | reverse | TTCGTCTTCCCCGCCTAGTA |
| MsauOBP17 | forward | GTGAGCGCAGGAGAAGATCA |
|  | reverse | ATTTTCAGCGTGCACAACCC |
| MsauOBP18 | forward | TAGACGGCCAACACAAAGGG |
|  | reverse | ATGTGGTCAACTCGCGGAAT |
| MsauOBP19 | forward | ACCGGAGAAGTGAATGCTCG |
|  | reverse | CACCGTGTATGACTCCGAGG |
| MsauOBP20 | forward | GTGAACACGTTCCTCGGAGT |
|  | reverse | ATCACTTGCGCCTTTCTCCA |
| MsauOBP21 | forward | TGAAGTTCCTCGTTGCTGCT |
|  | reverse | GTTGCACATCGGGCATCAAA |
| β-actin | forward | ACCATCACACCCTGATGACG |
|  | reverse | GGCAGCAAAATGTGTGACGA |
|  |  |  |

| Table S3 Quality statistics of filtered Reads in transcriptome sequencing | | | | | | | |
| --- | --- | --- | --- | --- | --- | --- | --- |
| Sample | Total raw reads | N | Adapter | Low quality | Clean reads | Clean reads ratio(%) | Clean reads Q30(%) |
| Ante1 | 45,573,892 | 958,608 | 1,469,796 | 36 | 43,145,452 | 94.67 | 91.27 |
| Ante2 | 45,573,892 | 851,820 | 1,319,574 | 42 | 43,402,456 | 95.24 | 91.28 |
| Ante3 | 45,573,892 | 1,102,968 | 1,949,326 | 42 | 42,521,556 | 93.30 | 91.36 |
| Average | 45,573,892 | 971,132 | 1,579,565 | 40 | 43,023,155 | 94.40 | 91.30 |

| Table S4 The quality indicators of unigenes after Denovo assembly | | | | | | | |
| --- | --- | --- | --- | --- | --- | --- | --- |
| Sample | Total Number | Total Length | Mean Length | N50 | N70 | N90 | GC(%) |
| Ante1 | 58,590 | 95,226,269 | 1,625 | 2,981 | 1,908 | 733 | 38.13 |
| Ante2 | 51,500 | 110,721,181 | 2,149 | 4,153 | 2,682 | 1,089 | 37.96 |
| Ante3 | 50,156 | 126,129,076 | 2,514 | 4,626 | 3,093 | 1,401 | 38.05 |
| All-Unigene | 77,444 | 184,461,623 | 2,381 | 4,540 | 2,951 | 1,263 | 38.03 |

| Table S5 Functional annotation results of Unigenes | | | | | | | | | | |
| --- | --- | --- | --- | --- | --- | --- | --- | --- | --- | --- |
| Values | Total | NR | NT | Swissprot | KEGG | KOG | Pfam | GO | Intersection | Overall |
| Number | 77,444 | 53,991 | 63,871 | 42,868 | 47,037 | 42,052 | 43,002 | 17,258 | 11,580 | 67,363 |
| Percentage | 100% | 69.72% | 82.47% | 55.35% | 60.74% | 54.30% | 55.53% | 22.28% | 14.95% | 86.98% |
|  |  |  |  |  |  |  |  |  |  |  |
